# Decision-making changes for patients and medical personnel in the management of acute appendicitis during the COVID-19 pandemic

**DOI:** 10.1186/s12873-022-00727-0

**Published:** 2022-10-24

**Authors:** Xuan Cai, Jingtao Bi, Zhixue Zheng, Yaqi Liu

**Affiliations:** Department of General Surgery, Beijing Jishuitan Hospital, 31 Xinjiekou East Street, Xicheng District, 100035 Beijing, China

**Keywords:** COVID-19, Acute Appendicitis, Emergency Treatment, Decision-making, Laparoscopic surgery

## Abstract

**Background:**

Acute appendicitis is the most common cause of acute abdomen. During the pandemic, to contain the spread of COVID-19, there were some integral changes in the medical processes based on the pandemic prevention policy, especially regarding emergency surgery. This study was conducted to investigate whether this pandemic also impacted the decision-making for both patients and medical personnel along with the treatment outcomes.

**Methods:**

Patients of age 18 years or older who were diagnosed clinically and radiologically with acute appendicitis between Jan 1, 2017, and Dec 31, 202,0 were reviewed. The data of 1991 cases were collected and used for this study. Two groups were formed, one group before and the other group after the outbreak. The gathered data included gender, age, appendiceal fecalith, outcomes of treatment, and long-term outcomes of non-operation (8 months follow-up). We also collected details of surgical cases from the above two groups. This data also included age, gender, appendiceal fecalith, fever, jaundice, length of onset before presenting to an emergency department (ED), anesthesia, surgery, white cell count, pathology, complications, and length of stay. We compared the above data respectively and analyzed the differences.

**Results:**

Compared to the period before the outbreak, patient visits for acute appendicitis remarkably dropped (19.8%), but surgical cases showed no change (dropped by roughly 5%). There were significant differences (P < 0.05) in failure of non-operation(after the pandemic 8.31% vs. before pandemic 3.22%), interval appendectomy(after pandemic 6.29% vs. before pandemic 12.84%), recurrence(after pandemic 23.27% vs. before pandemic 14.46%), and outcomes of recurrence. There was a significant difference (P < 0.05) in anesthesia method, surgery way, and complications( before pandemic 4.15% vs. after pandemic9.89% P < 0.05) in patients who underwent the surgery. There was no statistical difference (P > 0.05) concerning age, gender, fever, jaundice, appendiceal fecalith, white cell count, and length of onset before presenting to the ED.

**Conclusion:**

The current pandemic prevention policy is very effective, but some decision-making processes of doctor-patient have changed in the context of COVID-19 pandemic, that further influenced some treatment outcomes and might lead to a potential economic burden. It is essential to address the undue concern of everyone and optimize the treatment process.

## Background

The first case of the novel coronavirus (COVID-19) was confirmed in December 2019 in Wuhan, China. COVID-19 has eventually turned into a global pandemic that is still spreading around the world. Several measures were implemented in various countries and regions to effectively contain the outbreak, and they were regularly altered as the pandemic changed. In China, this disease was specified as a Class B infectious disease by the Law of the People’s Republic of China on the Prevention and Treatment of Infectious Diseases and was regarded as class A infectious disease to warrant anti-epidemic measures. All these indeed blocked the spread of the virus, especially in China, but they also resulted in a change in the overall healthcare strategy with a tremendous impact on the healthcare system. There is little doubt that emergency surgeons are facing unprecedented challenges in this situation [1].

Acute appendicitis is a common disease in acute surgical conditions with a lifetime incidence rate as high as 7–8% [2, 3]. While numerous studies demonstrate that non-operative management with antibiotic therapy is a safe option for people with acute uncomplicated appendicitis [4, 5], appendicectomy is still the primary management option [6, 7]. There have been multiple studies that reveal and share the experience of performing general surgery, including appendicectomy, as part of regular epidemic prevention and control. The related anti-epidemic process is becoming increasingly mature [8–10]. Beijing Jishuitan Hospital is a large comprehensive medical institution located in Beijing, the capital of China, which has been hit by this outbreak several times, since February 2020. Clinical activities have been carried out under regular epidemic prevention and control, which is a fact we must accept at present.

There have been some investigations about the changes in treatment behavior of people with acute appendicitis [8, 11, 12]. However, to improve therapy in the post-pandemic era, we still need to discover and collect additional information, particularly about some of the effects on decision-making by both patients and medical personnel in the course of managing acute appendicitis. Meanwhile, a long-term follow-up after conservative treatment is essential. The above can help us further comprehensively evaluate the impact of this pandemic on appendicitis patients. This study attempted to review and gather the information and data of patients diagnosed with acute appendicitis and treated in Beijing Jishuitan Hospital. Detecting and investigating this effect will help direct practices for the rest of the COVID-19 epidemic and possibly beyond.

## Materials and methods

**Fig. 1:**
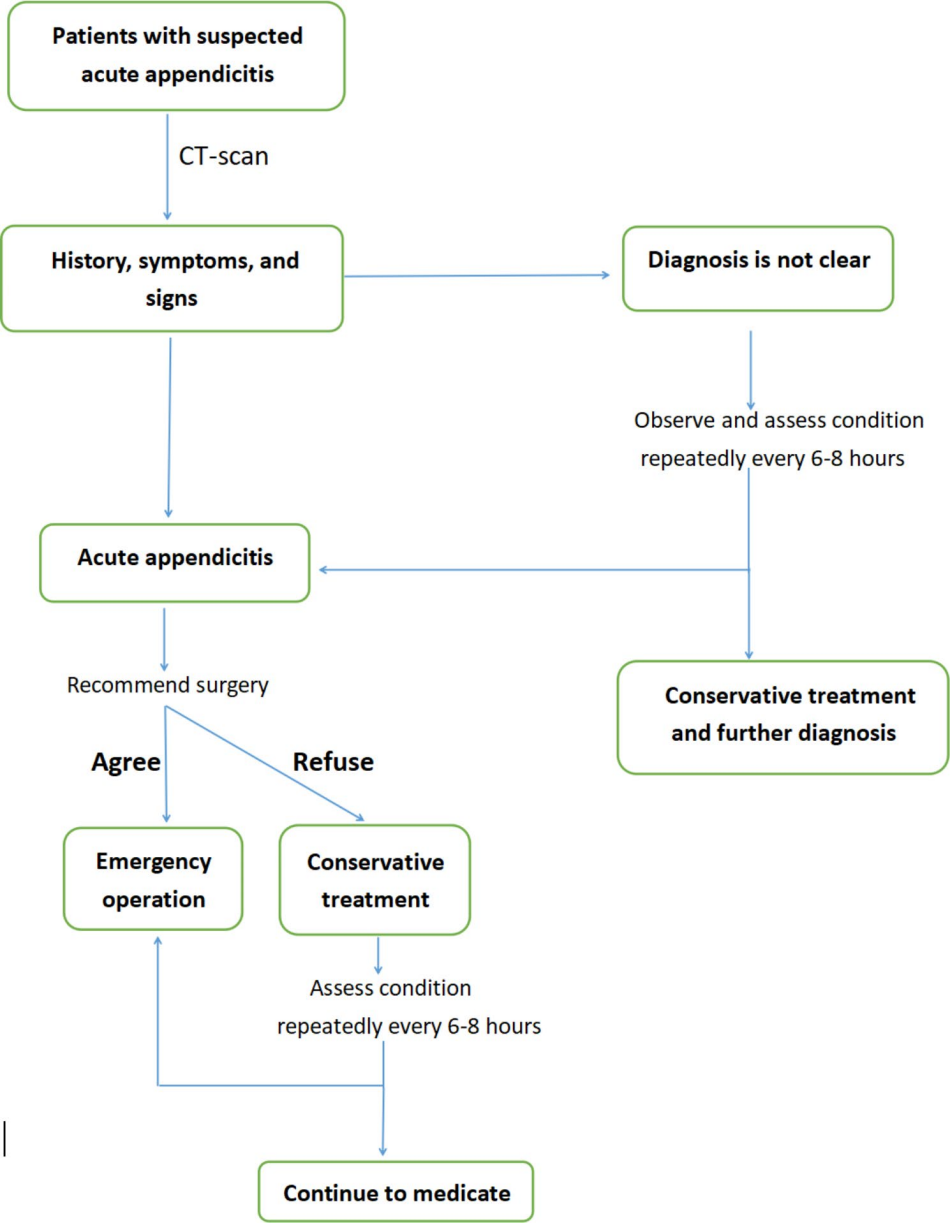
Algorithm for diagnosis and treatment of adult patients with suspected acute appendicitis

After the COVID-19 breakout in Wuhan, China, several epidemic prevention policies were established and implemented immediately in our hospital. According to the policy, every patient in the emergency department (ED) with a fever or epidemiological contact history has to undergo scanning for COVID-19. If the patient was required to be treated in emergency observation wards by a doctor, a Novel Coronavirus nucleic acid test must be done. Patients who were scheduled for emergency surgery were also administered a low-dose lung CT scan and a Novel Coronavirus antibody test. The patient would subsequently be treated under the appropriate pandemic protection level based on the results of these tests. Figure 1 illustrates the above process in a simplified sequence flow diagram (Fig. 1). Acute appendicitis was considered present when the appendiceal diameter exceeded 6 mm with wall thickening and at least one of the following was present: (1) abnormal contrast enhancement of the appendiceal wall, (2) inflammatory edema, or (3) fluid collections around the appendix [13, 14]. There have been numerous studies and guidelines that have concluded that non-operative treatment with antibiotics as the initial treatment for patients with uncomplicated acute appendicitis is a feasible alternative. Our hospital, on the other hand, adhered to the traditional principle and recommended an appendectomy as the primary treatment for patients showing symptoms for less than 72 h, though this was not mandatory. Meanwhile, patients with at least one of the following symptoms or signs were strongly recommended to undergo surgery: fever, shiver, perforation, or diffuse peritonitis. The non-operative approach was used when patients explicitly refused surgery. In this situation, the patient was observed in the emergency ward and critically assessed repeatedly every 6–8 h. Once the patient’s condition progressed, laparotomy was performed immediately. The criteria included aggravation of abdomen signs or symptoms, white cell count, or temperature rise. Patients with most of the acute inflammation resolved were also advised to have an interval appendectomy 6–8 weeks later.

**Fig. 2:**
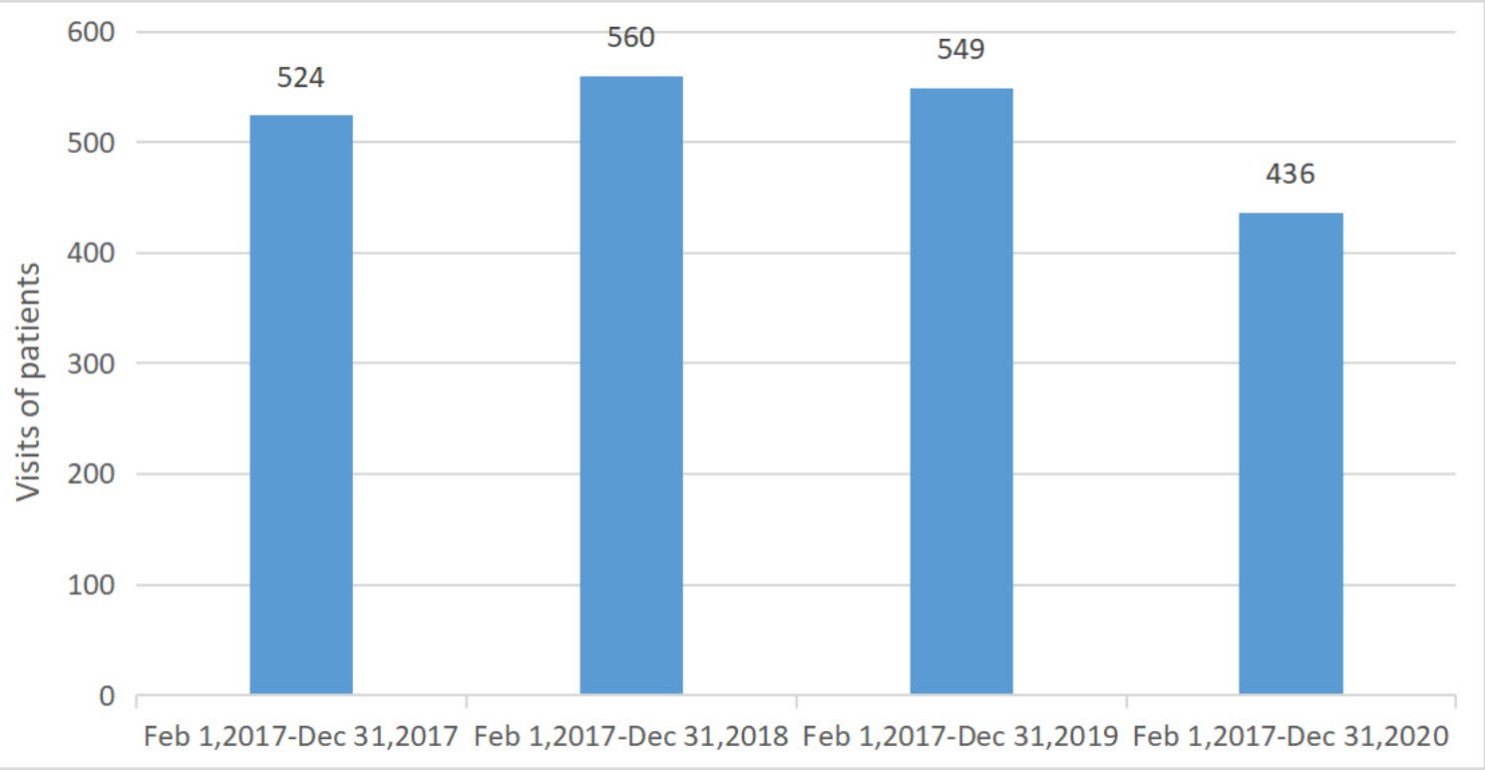
Visits of patients with acute appendicitis from February to December in 2017, 2018, 2019, and 2020

We retrospectively collected clinical data of all patients who were diagnosed with acute appendicitis and were treated in Beijing Jishuitan hospital during two time periods, one from January 1, 2017, to December 31, 201,9 and the second from February 1, 2020 to December 31, 2020. Inclusion criteria: (1) The diagnosis of acute appendicitis was confirmed by a CT scan of the abdomen; (2) The age of patients was at least 18 years; (3) Patients without any serious complications, ASA classification I or II. Exclusion criteria: (1) Patients with age less than 18 years; (2) Patients undergoing other surgery during the appendectomy; (3) Pregnant females with the appendix. There were 1740 reported cases of appendicitis in the period between January 1, 2017 and December 31, 2019, and 436 cases were included in this study from February 1, 2020 to December 31, 2020. The variation in the pattern of patient visits who were diagnosed as having acute appendicitis in ED is depicted in Fig. 2. To obtain the information and details about the treatment during the acute phase of the appendix, all these visitors were followed up after 8 months by telephonic interview. This follow-up also facilitated analysis of recurrence, interval surgery, and outcomes of recurrence for patients who received non-operation. Therefore, in this study, we do not only collect the clinical information of these patients with acute appendicitis patients, but also get more data of the long-term outcomes of non-surgery patients. Finally, there were 1582 cases with complete follow-up data during the period between January 1, 2017 and December 31, 2019. The success rate of follow-up was 90.92%, and 289 underwent an appendectomy. For the data of 409 cases, collected between February 1, 2020 and December 31, 2020, the success rate of follow-up was 93.81%. Ninety-one patients out of them were treated by surgery. There were two surgical options in this study, laparoscopic and traditional open methods.

The above cases with the integrity of data were divided into two groups. One was the post-pandemic group, which consisted of 409 cases from the period between February 1, 2020 and December 31, 2020. The other was the pre-pandemic group, which consisted of 1582 cases over 3 years (2017–2019). Patient’s medical records including gender, age, imaging, laboratory results, appendiceal fecalith, operative details, and pathology results were extracted from the chart. Meanwhile, we also gathered details of the treatment process through telephonic follow-up (Table 1). The criteria of non-operative management failure were based on the worsening of the signs and symptoms of patients who were treated by a non-surgical approach first followed by a surgical approach. The long-term prognosis of patients who were cured by antibiotics in their acute phase is shown in Table 2. There were 380 patients treated for appendectomy, and 91 of them were from the post-pandemic surgery group, while 289 were from the pre-pandemic group. We gathered data on the operation for these patients, including time of onset before the visit, white blood cell count, temperature, liver function, operation method, anesthesia mode, time of stay in the hospital, pathology, and complications as shown in Table 3.

**Table 1:** Comparison of treatment before and after the pandemic

	Pre-pandemic group	Post-pandemic group	P-value
**Cases**	1582	409	
**Age**			0.304
< 60years	1296(81.9%)	326(79.71%)	
> 60years	286(18.1%)	83(20.29%)	
**Appendiceal fecalith**			0.247
Yes	361(22.82%)	105(27.87%)	
No	1221(77.18%)	304(72.13%)	
**Gender**			0.359
Male	864(54.61%)	213(52.08%)	
Female	718(45.39%)	196(47.92%)	
**Treatment**			0.022^*^
Operation	238(15.04%)	57(13.94%)	
Non-operation	1293(81.73%)	318(77.75%)	
Failure of non-operation^a^	51(3.22%)	34(8.31%)	

*: P<0.05, statistically significant

a: The criteria of non-operative management failure were based on worsening of the signs and symptoms of patients who were treated by a non-surgical approach first followed by a surgical approach.

**Table 2:** Long-term outcomes of non-operation before and after the pandemic

	Pre-pandemic group	Post-pandemic group	P-value
**Non-operation**	1293	318	
**outcomes(8 months)**			0.003^*^
Interval appendectomy	166(12.84%)	20(6.29%)	
No recurrence	940(72.7%)	224(70.44%)	
recurrence	187(14.46%)	74(23.27%)	
**Outcomes of recurrence**	187	74	0.035^*^
Operation	74(39.57%)	19(25.68%)	
Non-operation	113(60.43%)	55(74.32%)	

*: P<0.05, statistically significant

**Table 3:** Comparison of operative treatment before and after the pandemic

	Pre-pandemic group	Post-pandemic group	P*-*value
**Cases**	289	91	
**Age**			0.373
< 60years	252(87.2%%)	76(83.52%)	
> 60years	37(12.8%)	15(16.48%)	
**Gender**			0.288
Male	180(62.28%)	51(56.04%)	
Female	109(37.72%)	40(43.96%)	
**Appendiceal fecalith**			0.194
Yes	185(64.01%)	65(71.43%)	
No	104(35.99%)	26(28.57%)	
**Fever(>37.5 °C)**			0.259
Yes	29(10.03%)	13(14.29%)	
No	260(89.97%)	78(85.71%)	
**Jaundice**			0.505
Yes	129(44.64%)	37(40.66%)	
No	160(55.36%)	54(59.34%)	
**Anesthesia**			0.005^*^
Lumbar anesthesia	84(29.07%)	41(45.25%)	
General anesthesia	205(70.93%)	50(54.95%)	
**Surgery**			
OA	89(30.8%)	49(53.85%)	0.007^*^
LA	200(69.2)	42(46.15%)	
**Conversion to open surgery**	1	0	
**Length of onset before presenting to ED(h)**	20.91 ± 17.06	21.01 ± 26.45	0.972
**White cell count(10** ^**9**^ **/L)**	14.14 ± 3.88	15.08 ± 3.51	0.096
**Pathology**			0.016*
Gangrene	79(27.34%)	37(40.66%)	
Non-gangrene	210(72.66%)	54(59.34%)	
**Complications**			0.037^*^
Yes	12(4.15%)	9(9.89%)	
No	277(95.85%)	82(90.11%)	
**Length of stay(days)**	4.73	5.14	0.467

*: P<0.05, statistically significant

Abbreviations: emergency department (ED); novel coronavirus (COVID-19); open appendectomy (OA); laparoscopic appendectomy (LA)

### Statistical analysis

We used SPSS software version 19.0 (IBM Statistics) to analyze the data. Between-group comparisons for continuous variables were performed with Student’s t-test or Welch’s t-test, the latter if the variances differed significantly. Categorical variables were compared using the Pearsonχ^2^ test or Fisher’s exact test. Two-tailed P-values of 0.05 or less were considered statistically significant.

## Results

Post-pandemic, the number of visits to the ED of Beijing Jishuitan hospital dropped significantly. The overall reduction was about 19.8% compared to previous years. However, compared to pre-pandemic years, the number of patients who underwent an appendectomy in the acute phase showed no remarkable change (dropped by roughly 5%). There were no significant differences in gender, age, and appendiceal fecalith before and after the pandemic (P > 0.05). However, the treatment (P = 0.022) and the outcomes of non-operative therapy (P = 0.003) had changed significantly (P < 0.05). The number of patients who experienced failure of non-operative treatment had increased significantly compared to that in the pre-pandemic period (after pandemic 8.31% vs. before pandemic 3.22%). The overall rate of non-operation approach getting converted to surgery was 8.31%. We also observed that fewer patients with a successful non-operation treatment were willing to opt for an interval appendectomy in the post-pandemic period (before pandemic 12.84% vs. after pandemic 6.89%, P < 0.05). However, there was an increased recurrence rate in those patients (14.46% vs. 23.27%), and the preference for appendectomy as the preferred treatment in the acute setting was lower in patients with increased recurrence than in patients before the outbreak (39.57% vs. 25.68%, P < 0.05). While comparing the baseline characteristics and outcomes of the patients who underwent surgery in the ED before and after the pandemic, we found no statistical differences in age, gender, fever, appendiceal fecalith, jaundice, length of onset before presenting to ED, white cell count, and length of stay (P > 0.05). There were significant differences in the way of anesthesia and surgery, pathology, and complications (P < 0.05). The incidence rate of lumbar anesthesia and open appendectomy after the outbreak was higher than before. The complication rate in the post-pandemic group was also significantly higher than before (4.15% vs. 9.89% P < 0.05). All cases of post-operation complications were surgical site infections except one case of deep vein thrombosis of the lower extremity in the pre-pandemic group.

## Discussion

There are no signs that the pandemic is “winding down,“ but the healthcare system or hospitals are now prepared for such events. While measures and bills have been put in place to protect the surgical workforce from nosocomial infections by following current local guidelines, the “war” against this virus is expected to be long drawn. We know that the pandemic has not only affected the process of treatment for acute appendicitis deeply but has also changed the treatment decision-making leading to different outcomes. By far, there is few report on the impact of the pandemic on doctor-patient and the treatment of acute appendicitis based on a long-term follow-up. By comparing data before and after the outbreak, we found that the number of visits of appendicitis patients (roughly 19.8% drop) and the rate of interval appendectomy (pre-pandemic 12.84% vs. post-pandemic 6.29%) significantly declined, but the number of patients who underwent an emergency appendectomy showed no remarkable change (dropped by roughly 5%). The failure rate of non-operation was increased (3.22% vs. 8.31%). Several other studies have also reported similar results of a decrease in visits by patients diagnosed with acute appendicitis during this outbreak. However, long-term follow-up data are not available [15, 16]. When we focused on patients who underwent an emergency appendectomy, the data showed that the length of onset before presenting to ED (20.91 ± 17.06 h vs. 21.01 ± 26.45 h) was similar to the length of onset before the breakout. Therefore, we speculated that there must be a certain number of patients experiencing uncomplicated appendicitis and who opted to stay at home with or without antibiotics, those patients significantly contributed to the decrease in the number of visits. Due to no change in emergency operation numbers and the length of onset, most of them might have eventually recovered. This might have led to a significant increase in the percentage of cases of gangrenous perforative appendicitis (Table 3). This result further confirmed that the non-operation approach can be used as a safe and effective alternative for acute uncomplicated appendicitis. A new meta-analysis in the setting of COVID-19 from Professor Sameh Hany Emile also provided the same result, and the study by professor Vishal K. Patel even included some complicated cases [17, 18]. However, the failure rate of non-operation significantly increased in our study, and there might have been a few patients with acute complicated appendicitis who refused the surgery in the first instance and were treated by non-operative management. Therefore, our view remains conservative and that non-operation treatment should be used only in uncomplicated acute appendicitis. There is certainly more scope to further define conservative treatment during this pandemic.

In addition, People have concerns about contracting COVID-19 by visiting hospitals, and more or less some fear, anxiety and stress by strictly screening and repeated epidemics. To reduce the probability of exposure to this virus, people avoided coming to the hospital or being hospitalized and prefer to take a wait-and-see attitude toward interval appendectomy. Therefore, we could find that the number of visits for interval appendectomy was lower in the post-pandemic period than in the pre-pandemic period (12.84% vs. 6.29%), and the rate of emergency surgery for recurrences was also lower than before (39.57% vs. 25.68%, P < 0.05) in our study. Meanwhile, that also showed a remarkably increased terecurrence rate (23.27% vs. 14.46%) in patients with acute appendicitis 8 months after the treatment with the non-operation approach. Certainly, we can not rule out the factor of make-decision change for patients that resulted in a significantly increase in the failure rate of non-operation. However, under the effective pandemic prevention policy, there was no reported case of nosocomial infection from our hospital during the pandemic. In any case, we did not anticipate an ideal outcome. The above results also confirmed that the pandemic influenced the decision-making of patients and created an obstacle between hospitals and patients. Despite many studies favoring non-operative management, recent studies have shown that the increase in recurrence rate within 1 year after the first non-surgical treatment for acute appendicitis cannot be ignored. The five-year follow-up result from the famous APPAC trial showed that in the non-operative treatment group, the recurrence rate reached up to 32.8%, and most of these cases emerged during the period one year after initial treatment [19–21]. However, whether an interval appendectomy after effective non-operative therapy could be considered routine has sparked heated controversy. Routine interval surgery is preferred after non-operative management for acute appendicitis, and this treatment applies only to patients with recurrent symptoms [7, 22, 23]. Acute appendicitis is fatal and should not be overlooked. Our recommendations should not exacerbate the dangerous situation in which patients with the highest recurrence risk are in a worse situation. For patients with recurrence, conservative treatment has repeatedly aggravated the economic burden and increased the health risk. Obviously, these problems is even more prominent during the pandemic. Since the outbreak of COVID-19, the current anti-pandemic policy and measures have already shown their value for the guaranteed running of medical institutions. We should focus on strengthening the healthcare system for patients after their initial treatment. By optimizing the medical processes, we can dispel the fear of this infection and increase patients’ confidence about staying protected from the pandemic.

The same strategy was applied to medical workers. After analyzing the data of patients who underwent an appendectomy in our hospital before and after the outbreak, the result showed that there were significant differences in anesthesia method, surgery procedure, and complications. According to the measures of our hospital for COVID-19 control and prevention, every in-patient is needed to undergo a Novel Coronavirus nucleic acid test or antibody test and low dose lung CT scan before admission. Based on the test results and patient histories, we determined the protective class for the surgery. It is up to the surgeon and anesthesiologist to decide whether or not to open the airway during surgery. The number of lumbar anesthesia cases steeply increased. However, there was no positive COVID-19 case, and all patients who were admitted to the hospital were mandatorily ruled out for COVID-19. This result reflected that anesthesiologists preferred lumbar anesthesia over general anesthesia during the pandemic period as chances of viral exposure are considerably higher in the case of airway opening. The literature also supports the importance of aerosol transmission of infectious diseases [24]. Therefore, these factors could have led to a significant decrease in the number of laparoscopic appendectomies. After comparing the treatment outcome of the two periods, the rate of postoperative complications was significantly higher than that before the outbreak (4.5% vs. 9.89%; P < 0.05). With the development and popularization of the laparoscopic technique, several guidelines and specialists have recommended laparoscopic appendectomy as the primary treatment for acute appendicitis [7]. Many studies pointed out that compared to an open appendectomy, laparoscopic surgery leads to a desirable outcome, lower rate of complications, shorter postoperative recovery time, less postoperative pain, and scarring. There was no effect on the operation time [25, 26]. In our study, only one patient had to be converted to open surgery during LA. The number of patients who underwent open surgery increased in the post-pandemic era, and the rate of postoperative complications was also significantly higher than before, particularly the rate of surgical site infections. Of course, The increase in gangrenous perforative appendicitis (Table 3) also contributed to these negative outcomes [27]. Therefore, we could not conclude that the significant decrease in the number of laparoscopic appendectomies results in an increased incidence of complications, but it is at least a risk factor. Moreover, as seen in our previous analyses, the incidence of gangrenous perforative appendicitis also increased in the post-pandemic era. Meanwhile, professor Georgios Markides and Gaik S Quah report that LA is superior to open surgery for complicated appendicitis [25, 28]. As a result, given the current anti-pandemic environment, we should make use of laparoscopy to improve treatment outcomes and promote laparoscopic surgery as the first line of treatment for acute appendicitis. The safe and effective pandemic prevention and its influence on the decision of medical personnel are conflicted. More research on anti-pandemic methods will further enhance medical personnel’s confidence and increase the use of general anesthesia.

## Conclusion

In conclusion, the non-operative approach could be an option for acute appendicitis, which is now verified as a flexible and secure treatment approach. However, a thorough clinical examination is necessary to ensure that the surgical intervention may be completed quickly. COVID-19 has spread rapidly to transform into a pandemic and has affected not only our lifestyle but also the decision-making of patients and medical workers. Although these changes did not significantly increase the incidence of serious adverse events related to acute appendicitis, the increased recurrence rate and complications with decreased rates of surgery in patients with recurrent episodes will eventually lift the burden on the whole healthcare system. Under the current anti-pandemic policy, we should work to strengthen the faith and reduce the fear of people. Certainly, the increased vaccination coverage might improve the situation. Further studies on this topic should be pursued. In the context of anti-pandemic efforts, we need to obtain more comprehensive information regarding treatment strategies to ensure patient safety.

## Data Availability

The datasets used and/or analyzed during the current study are available from the corresponding author upon reasonable request.

## Abbreviations

ED: emergency department
COVID-19: novel coronavirus
OA: open appendectomy
LA: laparoscopic appendectomy

## References

[1] Cheeyandira A. The effects of COVID-19 pandemic on the provision of urgent surgery: a perspective from the USA. J Surg Case Rep. 2020. 2020(4): rjaa109. 10.1093/jscr/rjaa109 PMC7176477 32346470

[2] Stewart B, Khanduri P, McCord C (2014). Global disease burden of conditions requiring emergency surgery. Br J Surg.

[3] Hardin DM (1999). Acute appendicitis: review and update. Am Fam Physician.

[4] Salminen P, Paajanen H, Rautio T (2015). Antibiotic Therapy vs Appendectomy for Treatment of Uncomplicated Acute Appendicitis: The APPAC Randomized Clinical Trial. JAMA.

[5] Abbo O, Trabanino C, Pinnagoda K (2018). Non-operative Management for Uncomplicated Appendicitis: An Option to Consider. Eur J Pediatr Surg.

[6] Flum DR, Davidson GH, Monsell SE (2020). A Randomized Trial Comparing Antibiotics with Appendectomy for Appendicitis. N Engl J Med.

[7] Di Saverio S, Podda M, De Simone B (2020). Diagnosis and treatment of acute appendicitis: 2020 update of the WSES Jerusalem guidelines. World J Emerg Surg.

[8] Kumaira Fonseca M, Trindade EN, Costa Filho OP, Nácul MP, Seabra AP (2020). Impact of COVID-19 Outbreak on the Emergency Presentation of Acute Appendicitis. Am Surg.

[9] Zhou Y, Cen LS (2020). Managing acute appendicitis during the COVID-19 pandemic in Jiaxing, China. World J Clin Cases.

[10] Javanmard-Emamghissi H, Boyd-Carson H, Hollyman M (2021). The management of adult appendicitis during the COVID-19 pandemic: an interim analysis of a UK cohort study. Tech Coloproctol.

[11] Baral S, Chhetri RK, Thapa N (2021). Comparison of acute appendicitis before and within lockdown period in COVID-19 era: A retrospective study from rural Nepal. PLoS ONE.

[12] Finkelstein P, Picado O, Muddasani K (2021). A Retrospective Analysis of the Trends in Acute Appendicitis During the COVID-19 Pandemic. J Laparoendosc Adv Surg Tech A.

[13] Lai V, Chan WC, Lau HY, Yeung TW, Wong YC, Yuen MK (2012). The diagnostic power of various computed tomography signs in diagnosing acute appendicitis. Clin Imaging.

[14] Kim K, Kim YH, Kim SY (2012). Low-dose abdominal CT for evaluating suspected appendicitis. N Engl J Med.

[15] Tankel J, Keinan A, Blich O (2020). The Decreasing Incidence of Acute Appendicitis During COVID-19: A Retrospective Multi-centre Study. World J Surg.

[16] Orthopoulos G, Santone E, Izzo F (2021). Increasing incidence of complicated appendicitis during COVID-19 pandemic. Am J Surg.

[17] Emile SH, Hamid H, Khan SM, Davis GN (2021). Rate of Application and Outcome of Non-operative Management of Acute Appendicitis in the Setting of COVID-19: Systematic Review and Meta-analysis. J Gastrointest Surg.

[18] Patel VK, Ye K, In H, Scheinfeld MH (2021). Non-operative Management for Acute Appendicitis During the COVID-19 Pandemic Does Not Increase the Rate of Complications. J Gastrointest Surg.

[19] Salminen P, Tuominen R, Paajanen H (2018). Five-Year Follow-up of Antibiotic Therapy for Uncomplicated Acute Appendicitis in the APPAC Randomized Clinical Trial. JAMA.

[20] Sippola S, Grönroos J, Sallinen V (2018). A randomised placebo-controlled double-blind multicentre trial comparing antibiotic therapy with placebo in the treatment of uncomplicated acute appendicitis: APPAC III trial study protocol. BMJ Open.

[21] Vons C, Barry C, Maitre S (2011). Amoxicillin plus clavulanic acid versus appendicectomy for treatment of acute uncomplicated appendicitis: an open-label, non-inferiority, randomised controlled trial. Lancet.

[22] Al-Kurd A, Mizrahi I, Siam B (2018). Outcomes of interval appendectomy in comparison with appendectomy for acute appendicitis. J Surg Res.

[23] Hayes D, Reiter S, Hagen E (2021). Is interval appendectomy really needed? A closer look at neoplasm rates in adult patients undergoing interval appendectomy after complicated appendicitis. Surg Endosc.

[24] Tang S, Mao Y, Jones RM (2020). Aerosol transmission of SARS-CoV-2? Evidence, prevention and control. Environ Int.

[25] Quah GS, Eslick GD, Cox MR (2019). Laparoscopic appendicectomy is superior to open surgery for complicated appendicitis. Surg Endosc.

[26] Güler Y, Karabulut Z, Çaliş H, Şengül S (2020). Comparison of laparoscopic and open appendectomy on wound infection and healing in complicated appendicitis. Int Wound J.

[27] Emile SH, Elfallal AH, Elbaz SA, Elmetwally AM (2021). Development and validation of risk prediction score for incisional surgical site infection after appendectomy. Updates Surg.

[28] Markides G, Subar D, Riyad K (2010). Laparoscopic versus open appendectomy in adults with complicated appendicitis: systematic review and meta-analysis. World J Surg.

